# Investigation on Transparent, Conductive ZnO:Al Films Deposited by Atomic Layer Deposition Process

**DOI:** 10.3390/nano12010172

**Published:** 2022-01-05

**Authors:** Kai Zhao, Jingye Xie, Yudi Zhao, Dedong Han, Yi Wang, Bin Liu, Junchen Dong

**Affiliations:** 1 School of Information & Communication Engineering, Beijing Information Science and Technology University, Beijing 100101, China; zhaokai@bistu.edu.cn (K.Z.); jingyexie@bistu.edu.cn (J.X.); zhaoyd@bistu.edu.cn (Y.Z.); 2 Institute of Microelectronics, Peking University, Beijing 100871, China; imewangyi@pku.edu.cn; 3 Systems Engineering Institute, Academy of Military Sciences, Beijing 100191, China; dalizdalizi@163.com

**Keywords:** transparent electrodes, atomic layer deposition process, Al-doped ZnO films, thin film transistors

## Abstract

Transparent electrodes are a core component for transparent electron devices, photoelectric devices, and advanced displays. In this work, we fabricate fully-transparent, highly-conductive Al-doped ZnO (AZO) films using an atomic layer deposition (ALD) system method of repeatedly stacking ZnO and Al_2_O_3_ layers. The influences of Al cycle ratio (0, 2, 3, and 4%) on optical property, conductivity, crystallinity, surface morphology, and material components of the AZO films are examined, and current conduction mechanisms of the AZO films are analyzed. We found that Al doping increases electron concentration and optical bandgap width, allowing the AZO films to excellently combine low resistivity with high transmittance. Besides, Al doping induces preferred-growth-orientation transition from (002) to (100), which improves surface property and enhances current conduction across the AZO films. Interestingly, the AZO films with an Al cycle ratio of 3% show preferable film properties. Transparent ZnO thin film transistors (TFTs) with AZO electrodes are fabricated, and the ZnO TFTs exhibit superior transparency and high performance. This work accelerates the practical application of the ALD process in fabricating transparent electrodes.

## 1. Introduction

Transparent electrodes combine high transmittance and low resistivity well, making them core components in the fields of photoelectric devices, transparent electron devices, advanced displays, and solar cells [1,2,3]. The representative transparent electrodes include transparent conductive oxide (TCO) films, graphene and carbon nanotubes, metal nanowires, metal meshes, and ultrathin metal films [4,5,6]. Among them, TCO films are the most widely utilized transparent electrodes due to their flexibility, high uniformity, large-scale fabrication, and the fact that they are a mature technology [7,8]. Typical TCO transparent electrodes are InSnO (ITO) films, which have been commercialized for more than three decades [9]. However, scarcity of the metal element In will inevitably cause a critical issues of rising cost and manufacture unsustainability [10]. Therefore, the development of low-cost TCO films play a role in maintaining the prevalence of them.

Al-doped ZnO (AZO) films are regarded as the successor of ITO films because of their extremely low cost as well as their comparable transmittance and resistivity with ITO films [11]. Magnetron sputtering is a commonly used process to deposit AZO films. So far, many articles on sputtering-deposited AZO films and correspondingly application examples have been reported [11,12,13,14]. However, the sputtering process suffers from the distinct disadvantages of high-vacuum background pressure, surface damage of pre-deposited films, worse repeatability, and film composition limitations [15,16]. Therefore, it is important to develop novel processes for depositing transparent conductive AZO films.

During the past decade, the atomic layer deposition (ALD) process has become a mature film-deposition method. Utilizing the layer-by-layer growth mode, the ALD process excellently combines high quality films with good repeatability [17]. Moreover, ALD-deposited films integrates atomic-level thickness controllability, wonderful conformality, and large-scale uniformity [18]. Recently, the ALD process has been of increasing interest in the fabrication of AZO films. Dimitrov et al. [19], Marques et al. [20], and Li et al. [21] studied ALD-deposited flexible AZO films with high transmittance and low resistance. Swatowska et al. [22], Li et al. [23], and Wu et al. [24] investigated Al concentration on the properties of ALD-deposited AZO films. Macco et al. [25] and Oh et al. [26] demonstrated application of the ALD-deposited AZO films to solar cells. Mundle et al. applied AZO films to memristor applications [27]. Even though ALD-deposited AZO films present broad prospects, the underlying mechanisms of current conduction of the AZO films are still not fully understood, and their application to transparent electron devices has not been verified.

In this work, fully-transparent, highly-conductive AZO films are deposited using an ALD system. Current conduction mechanisms of the ALD-deposited AZO films are examined by characterizing optical property, conductivity, material components, crystallinity, and surface morphology. We demonstrated high-performance, transparent ZnO thin film transistors TFTs, where the ZnO active layer, Al_2_O_3_ gate dielectric, and AZO electrodes are all deposited by the ALD process. This work paves the way for the practical utilization of the ALD process in transparent electrodes.

## 2. Materials and Methods

**Figure 1:**
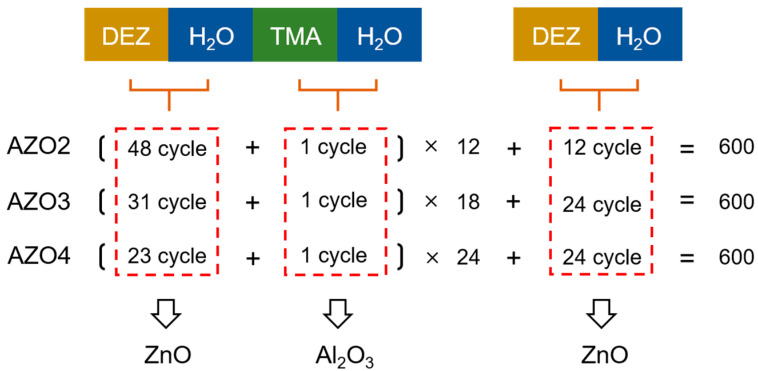
Schematic diagram of fabrication methods for AZO films. Total cycle number of AZO films are 600.

The AZO films were deposited on amorphous glass substrates and single-crystal silicon substrates (<100>, 1–5 Ω·cm, p-type) using an ALD system (M-150, MEZ, Finland) at 120 °C. The substrates were pre-cleaned in acetone, alcohol, and deionized water (H_2_O), respectively, using an ultrasonic cleaner. Detailed fabrication methods of the AZO films are shown in Figure 1. Three different AZO films were deposited by repeatedly stacking ZnO and Al_2_O_3_ layers. For the sake of simplicity, we named the AZO films AZO2, AZO3, and AZO4, respectively. The cycle number of all the AZO films are fixed at 600. The Al cycle ratios of the AZO films are 2, 3, and 4%, respectively. A 600-cycle ZnO film was deposited, and its Al cycle ratio was 0%. The precursors for the Al, Zn, and O elements were trimethylaluminum (TMA, 99.9999%, Nanjing Aimouyuan Scientific Equipment, Nanjing, China), diethylzinc (DEZ, 99.9999%, Nanjing Aimouyuan Scientific Equipment, Nanjing, China), and H_2_O, respectively. Pure N_2_ (99.9999%) was employed as a carrier as well as a purge gas, and purge time was set as 25 s.

Before measurement, the AZO films were thermally annealed at 200 °C in vacuum for 1 h. Transmittance was characterized by the transmittance spectrum (Omni-λ500, Zolix, Beijing, China). Electron concentration and resistivity were characterized by Hall measurement (HMS-3000, Ecopia, Anyang, South Korea). Material components were characterized by X-ray photoelectron spectroscopy (XPS, Axis Ultra, Kratos, Manchester, United Kingdom). Lattice structure was characterized by X-ray diffraction (XRD, D/MAX 2000, Rigaku, Tokyo, Japan) and transmission electron microscope (TEM, Tecnai F20, FEI, Hillsboro, United States). Surface morphology was characterized by atomic force microscope (AFM, Dimension Icon, Bruker, Billerica Massachusetts, United States; Tips model, Scan ASYST-Air). Electron concentration, resistivity, transmittance spectra, and XRD spectra were obtained by characterizing the ZnO and AZO films on glass substrates. TEM images, AFM images, and XPS spectra were obtained by characterizing the ZnO and AZO films on silicon substrates.

## 3. Results and Discussion

### 3.1. Resistance and Transmittance of AZO Films

**Figure 2:**
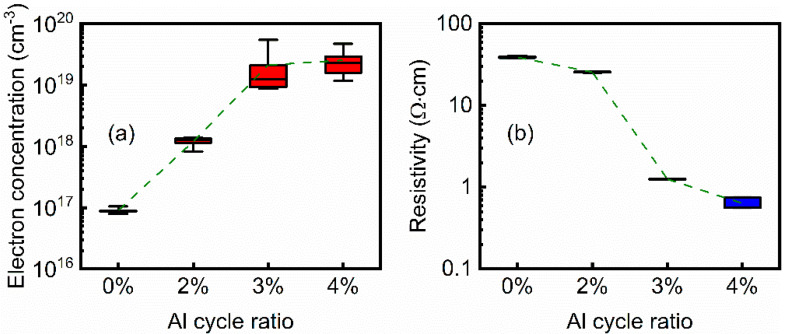
(**a**) Electron concentration of ZnO and AZO films. (**b**) Resistivity of ZnO and AZO films. *n* = 5. ZnO and AZO films were deposited on glass substrates.

To determine the electrical properties of the ZnO and AZO films, Hall measurement was performed. As shown in Figure 2a, electron concentration presents an increase trend from about 10^17^ to 10^19^ cm^−3^ as Al cycle ratio increases from 0 to 3%. Thereafter, carrier concentration tends to be saturation as the Al cycle ratio further increases to 4%. Taking the ZnO film as the control group, it is possible to see that some of the Al atoms effectively play a role in the substitutional doping of the Zn atoms, but the excess Al atoms act as interstitial doping or exist in the form of aluminum oxide. This phenomenon is caused by the low doping efficiency of Al atoms in ZnO films [28]. Notably, electron concentration of the AZO films is comparable to that of the previously-reported ITO films [29,30,31]. Resistivity of the ZnO and AZO films are shown in Figure 2b. It was found that resistivity negatively correlates with the Al cycle ratio, suggesting that conductivity positively correlates with the Al cycle ratio. These trends are consistent with the electron concentration result.

**Figure 3:**
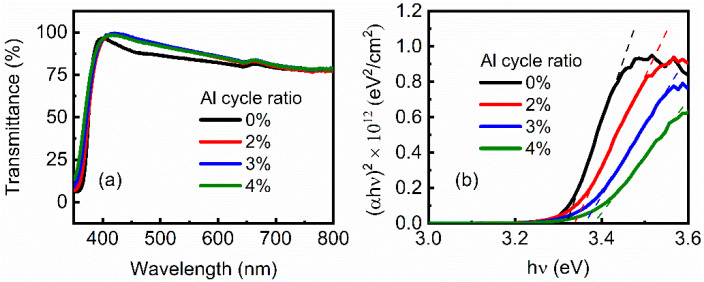
(**a**) Transmittance of ZnO and AZO films. (**b**) (αhν)^2^-hν curve of ZnO and AZO films. ZnO and AZO films were deposited on glass substrates.

The transmittance spectra of the ZnO and AZO films are shown in Figure 3a. In the visible light range, all the films show an average transmittance above 75%. Furthermore, the transmittance of the AZO films is higher than that of the ZnO film. According to transmittance spectra, the (αhν)^2^-hν curves of the ZnO and AZO films were depicted, where α, h, and ν are absorbance, Planck constant, and light frequency, respectively [32]. As shown in Figure 2b, optical bandgap width (E_opt_) can be obtained by extending the slope of the curves (dashed lines). It is shown that E_opt_ presents an increasing trend as Al cycle ratio increases. The main reason for this is that the incorporation of Al atoms in the ZnO films increases electron concentration and raises the Fermi level; therefore, absorbing edges shift toward the higher energies, leading to enlargement of E_opt_ and an increase of transmittance [33].

To sum up, the optimal AZO films exhibit a low resistivity of about 1 Ω·cm and a high transmittance above 75%, which are comparable to previous works [19,22,25]. Thus, the ALD-deposited AZO films in this work have a great potential to be used as transparent electrodes.

### 3.2. Crystallinity, Surface Morphology, and Material Components of AZO Films

**Figure 4:**
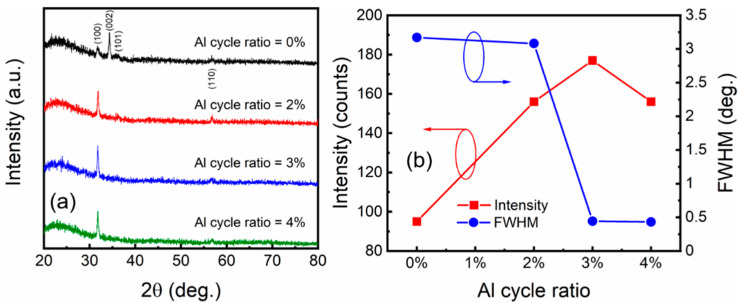
(**a**) XRD spectra of ZnO and AZO films. (**b**) Maximum height and FWHM of (100) peak of ZnO and AZO films. ZnO and AZO films were deposited on glass substrates.

To gain an insight into the crystallinity of the ZnO and AZO films, XRD measurement was performed, as shown in Figure 4a. For the ZnO film, the XRD spectrum reveals a hexagonal structure, showing up as a preferred growth along the c axis [22]. Obviously, the intensity of the (002) peak is much stronger than that of the other peaks, including the (100), (101), and (110) peaks. For the AZO film, the (002) peak fades away, while the (100) peak becomes dominant. The (101) and (110) show no evident changes. Therefore, Al doping induces preferred-growth-orientation transition from (002) to (100). Consequently, crystallization of local AZO grains is along the horizontal plane of the AZO films. In order to quantitatively analyze the XRD spectra, we extracted the detailed intensity and the full width at half maximum (FWHM) of the (100) peak of the ZnO and AZO films, as shown in Figure 4b. Intensity presents a trend of first increasing and then decreasing with Al cycle ratio; moreover, the AZO films achieve a relatively high crystallinity at an Al cycle ratio of 3%. FWHM presents a significant decrease as Al cycle ratio increases from 2% to 3%; it then keeps a small value of 0.43° as Al cycle ratio increases to 4%. Grain size (D) along the (100) orientation was then evaluated according to the Scherrer equation D = 0.9λ/(β·cosθ), where D, λ, θ, and β are grain size, X-ray wavelength, diffraction angle, and FWHM, respectively. The grain sizes of the ZnO and AZO films are shown in Table 1. The results show that local AZO grains reach a grain size of over 18 nm at an Al cycle ratio of 3%. 

**Figure 5:**
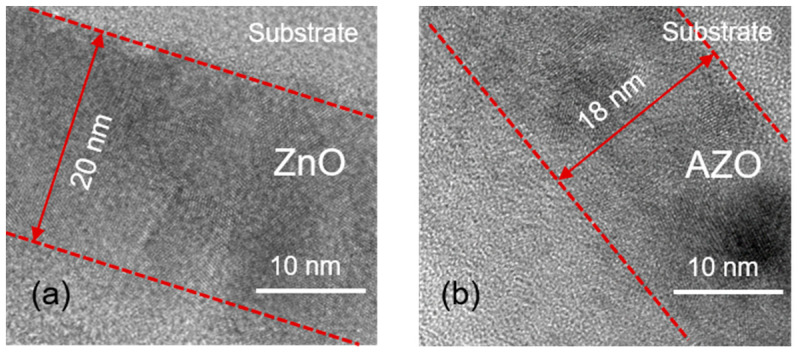
TEM images of a cross section of (**a**) ZnO film and (**b**) AZO film. Al cycle ratio of AZO film is 3%. ZnO and AZO films were deposited on silicon substrates.

TEM measurement was performed to study the lattice structure of the ZnO and AZO films. In order to observe microstructure and film thickness simultaneously, the cycle number of the ZnO and AZO film were scaled down to 150. As shown in Figure 5a,b, both the ZnO and AZO film exhibit a polycrystalline lattice structure, which is consistent with previously-reported works [34,35]. Noticeably, the thickness of the AZO film (18 nm) is thinner than that of the ZnO film (20 nm). Combined with the XRD results, it can be seen that preferred-growth-orientation transition of local AZO grains is the main reason for this phenomenon. For electron device applications, a (100) crystallization orientation of poly-crystalline AZO films is benefit for enhancing stability and mechanical performance [36].

**Figure 6:**
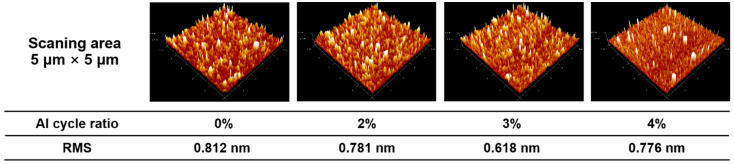
3D AFM images of ZnO and AZO films. Al cycle ratio and RMS are list below 3D AFM images. ZnO and AZO films were deposited on silicon substrates.

To examine the surface morphology of the ZnO and AZO films, AFM measurement was performed, as shown in Figure 6. The scanning area was set as 5 μm × 5 μm. The 3D AFM images reveal that all the films have a uniform and smooth surface. The root-mean-square (RMS) roughness of the ZnO and AZO films are summarized below the 3D AFM images. It was found that RMS roughness presents a trend of decreasing at first and then increasing with the Al cycle ratio, and a smaller RMS roughness of 0.618 nm is obtained at an Al cycle ratio of 3%. The transition of microstructure and crystallinity may be the underlying mechanism for the RMS roughness trend.

**Figure 7:**
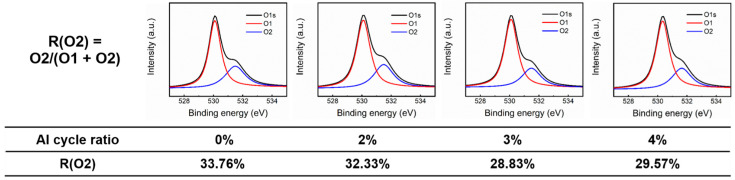
XPS spectra of ZnO and AZO films. Al cycle ratio and R(O2) are list below XPS spectra. ZnO and AZO films were deposited on silicon substrates.

To explore the material components of the ZnO and AZO films, XPS measurement was performed, as shown in Figure 7. Using Gaussian fitting, the O 1s spectra were deconvoluted into two individual peaks, representing the O^2-^ ions surrounded by metal atoms (O1, 530.10 eV) and the O^2-^ ions in oxygen deficient regions (O2, 531.49 eV), respectively [37,38]. The area ratios of the oxygen defects, i.e., R(O2) = O2/(O1 + O2), were extracted and are listed below the XPS spectra. R(O2) shows a dramatic decrease from 33.76% to 28.83% as the Al cycle ratio increases from 0% to 3%, and R(O2) slightly increases to 29.57% as the Al cycle ratio increases to 4%. Therefore, it can be seen that the Al atoms in the AZO films play a role in suppressing oxygen defects, and the AZO film with an Al cycle ratio of 3% has the least amount of oxygen defects.

By characterizing electrical properties and film properties, the current conduction mechanisms of the AZO films were examined. (1) The XRD spectra and TEM images confirm that the Al atoms in the AZO films induce preferred-growth-orientation transition from (002) to (100), i.e., crystallization orientation of the AZO films is along the horizontal plane. (2) Crystallization along the horizontal plane promotes the formation of flat surface of the AZO films, which weakens surface scattering and enhances current conduction across the AZO films. (3) Substitutional Al atoms in the AZO films suppress the generation of oxygen defects, thus improving the quality of AZO films and promoting the application of the AZO films to electron devices. (4) Synthesizing the above results, an optimal Al cycle ratio of AZO films is 3%.

### 3.3. Performance of Transparent ZnO TFTs with AZO Electrodes

**Figure 8:**
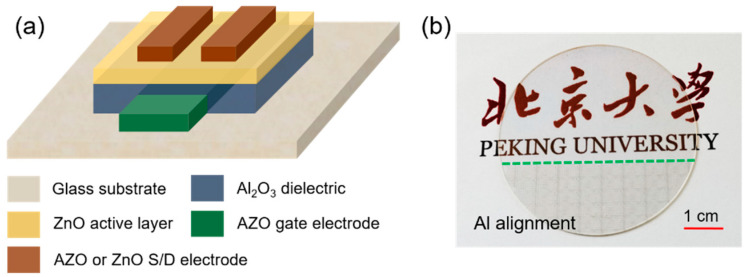
(**a**) Schematic device structure of ZnO TFTs. (**b**) Photo image of ZnO TFTs. ZnO and AZO films are used as S/D electrode.

ZnO TFTs with AZO electrodes were fabricated on glass substrate. Figure 8a shows the schematic device structure of the ZnO TFTs, of which ZnO, Al_2_O_3_, and AZO (Al cycle ratio of 3%) film were used as active layer, gate insulator, and gate electrode, respectively. Compared with previous works, the transparent ZnO TFTs in our work were fabricated entirely by the ALD process. Moreover, all the components employed were non-toxic, abundant, and low-cost materials. ZnO and AZO (Al cycle ratio of 2%, 3%, and 4%) films were used as source/drain (S/D) electrodes, and detailed fabrication processes were the same as in the Materials and Methods section. The feature size of the ZnO TFTs, defined by the S/D electrodes, is W/L = 100 µm/100 µm. Figure 8b shows a photographic image of the ZnO TFTs arrays. The region below the green dashed line includes the ZnO TFTs, along with Al marks, which were used for device alignment during the experiment, while the region above the green dash line includes the ZnO TFTs arrays we are concerned with. Remarkably, the ZnO TFT arrays show excellent transparency, demonstrating the potential of the AZO films being used in transparent electron devices.

**Figure 9:**
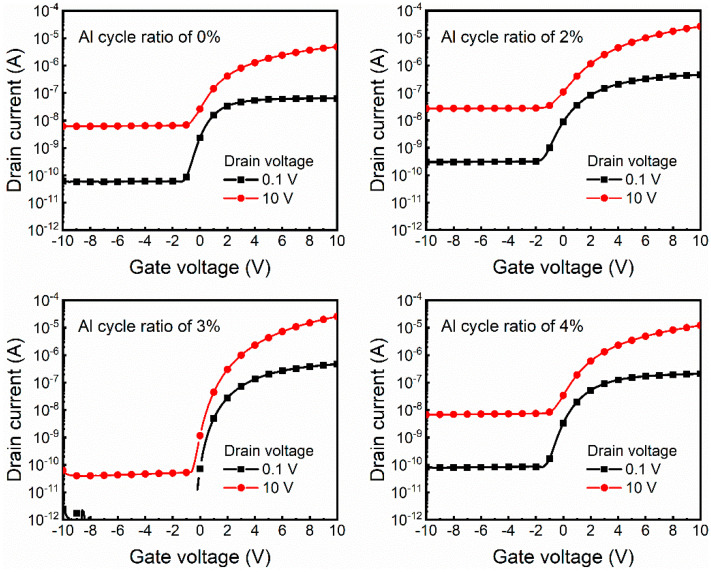
I_D_–V_G_ curves of ZnO TFTs. ZnO and AZO films are used as the S/D electrode.

The drain current–gate voltage (I_D_–V_G_) curves of the ZnO TFTs were measured, as shown in Figure 9. Notably, the ZnO TFTs with an AZO S/D electrode of 3% Al cycle ratio exhibit much better transfer characteristics than the other devices, manifesting a steep sub-threshold region and low off-state current. The major electrical parameters include a field-effect mobility (µ_FE_) of 4.07 cm^2^V^−1^s^−1^, a sub-threshold swing (SS) of 194.94 mV/decade, a turn-on voltage (V_ON_) of −0.2 V, and an on-to-off state current ratio (I_ON_/I_OFF_) of 7.39 × 10^5^. For the ALD-deposited AZO electrodes, the device performance of the ZnO TFTs has been significantly improved in this work [21]. Moreover, electrical parameters are comparable to devices with sputtering-deposited AZO electrodes [12,14]. The electrical parameters of all the devices are summarized in Table 2.

**Figure 10:**
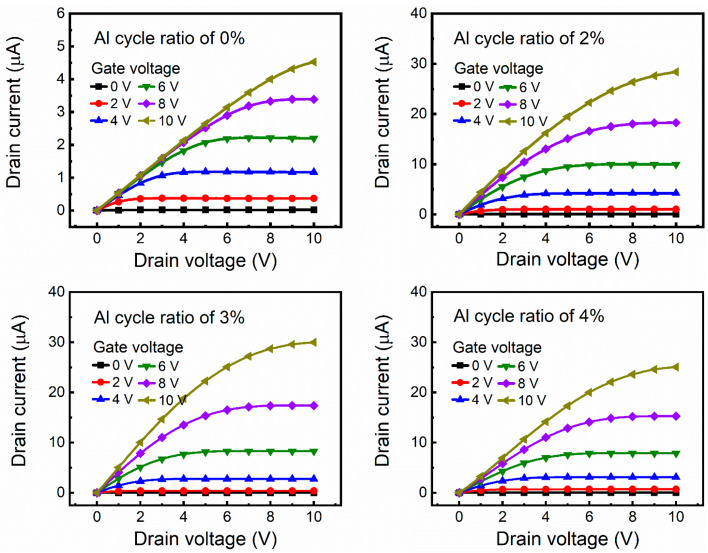
I_D_–V_D_ curves of ZnO TFTs. ZnO and AZO films are used as S/D electrode.

To evaluate contact property of the ZnO and AZO S/D electrodes, the drain current–drain voltage (I_D_–V_D_) curves of the ZnO TFTs were measured, as shown in Figure 10. All the I_D_–V_D_ curves show the apparent linear region and saturation region. We can see that the saturation current presents a trend of first increasing and then decreasing with Al cycle ratio, which may result from the oxygen components inducing contact-resistance differences. When the Al cycle ratio is 3%, the ZnO TFTs exhibit the best performance. Additionally, there is no current crowding phenomenon at the linear region, verifying ideal Ohmic contact between the ZnO active layer and the AZO S/D electrode. Noticeably, the electrical parameters of the ZnO TFTs are consistent with the film characterization results. To sum up, we illustrate the feasibility of ALD-deposited AZO films to serve as electrode materials for transparent electron devices.

## 4. Conclusions

In this work, transparent conductive AZO films were fabricated by the ALD process, and they were applied to ZnO TFTs as gate and S/D electrodes. By characterizing film properties, the current conduction mechanisms of the AZO films were analyzed. The results show that electron concentration and optical band gap width positively correlate with the Al cycle ratio of the AZO films, so that Al atoms not only reduces resistivity but also increases transmittance. The crystallinity results illustrate that Al atoms induce the crystallization transition of local grains from along c axis to along the horizontal plane, which improves surface morphology and enhances current conduction across the AZO films. Moreover, Al atoms suppress the oxygen defects of AZO films, consequently forming ideal Ohmic contact between the ZnO active layer and the AZO S/D electrode. Notably, the ZnO TFTs exhibit superior transparency and high performance. This work demonstrates the broad prospects of transparent AZO films and paves the way for the prevalence of the ALD process in the field of transparent electrodes.

## Figures and Tables

**Table 1:** Grains size of ZnO and AZO films. (100) orientation.

Al Cycle RATIO (%)	0	2	3	4
**D (nm)**	2.61	2.68	18.61	19.16

**Table 2:** Electrical parameters of ZnO TFTs. Al cycle ratios of AZO S/D electrodes are 0%, 2%, 3%, and 4%, respectively.

Al Cycle Ratio (%)	µ_FE_ (cm^2^V^−1^s^−1^)	SS (mV/decade)	V_ON_ (V)	I_ON_/I_OFF_
0	1.07	627.97	−1.3	1.08 × 10^3^
2	3.64	960.93	−1.9	1.46 × 10^3^
3	4.07	194.94	−0.2	7.34 × 10^5^
4	2.29	696.54	−1.5	2.55 × 10^3^

## Data Availability

The data that support the findings of this study are available from the corresponding author upon reasonable request.
